# The contribution of DNA repair pathways to genome editing and evolution in filamentous pathogens

**DOI:** 10.1093/femsre/fuac035

**Published:** 2022-07-09

**Authors:** Jun Huang, David E Cook

**Affiliations:** Department of Plant Pathology, Kansas State University, 1712 Claflin Road, Throckmorton Hall, Manhattan, KS 66506, United States; Department of Plant Pathology, Kansas State University, 1712 Claflin Road, Throckmorton Hall, Manhattan, KS 66506, United States

**Keywords:** DNA double-strand break repair, CRISPR-Cas, microhomology-mediated end joining, two-speed genome, filamentous pathogens, Biased variation

## Abstract

DNA double-strand breaks require repair or risk corrupting the language of life. To ensure genome integrity and viability, multiple DNA double-strand break repair pathways function in eukaryotes. Two such repair pathways, canonical non-homologous end joining and homologous recombination, have been extensively studied, while other pathways such as microhomology-mediated end joint and single-strand annealing, once thought to serve as back-ups, now appear to play a fundamental role in DNA repair. Here, we review the molecular details and hierarchy of these four DNA repair pathways, and where possible, a comparison for what is known between animal and fungal models. We address the factors contributing to break repair pathway choice, and aim to explore our understanding and knowledge gaps regarding mechanisms and regulation in filamentous pathogens. We additionally discuss how DNA double-strand break repair pathways influence genome engineering results, including unexpected mutation outcomes. Finally, we review the concept of biased genome evolution in filamentous pathogens, and provide a model, termed Biased Variation, that links DNA double-strand break repair pathways with properties of genome evolution. Despite our extensive knowledge for this universal process, there remain many unanswered questions, for which the answers may improve genome engineering and our understanding of genome evolution.

## Teaser

This review summarizes and compares the molecular mechanism, hierarchy, and regulation of four DNA double-strand break repair pathways in animal and fungal models, with the aim to connect these DNA repair pathways to genome engineering outcomes and biased genome evolution in filamentous pathogens.

## Introduction

The last 75 years have produced the structure of DNA, the birth of biotechnology, the development of high-throughput sequencing and advanced computational power, fueling discovery and understanding of the genome. This has led to an understanding of DNA synthesis, usage, and repair, and the ability to precisely manipulate DNA to alter life. This has revolutionized our understanding of biology.

One area of DNA biology that has received significant attention is how cells repair DNA that has undergone damage. There are a number of ways that DNA can be damaged (Chatterjee and Walker 2017) and specific repair mechanisms have evolved to fix or respond to the different types of physiochemical DNA damage, generally referred to as the DNA-damage response (Jackson and Bartek 2009). This review focuses on one specific type of DNA damage, termed DNA double-strand breaks (DSBs), in which the covalent phosphodiester bond between adjacent sugar atoms of DNA nucleotides are broken on both DNA strands. Repairing DNA DSBs is critical for cell viability and genome integrity, and cells cannot undergo replication and division with such lesions (Ceccaldi et al. 2016). While repair is a critical process, it is also dangerous to the cell, in that improper DNA rejoining can result in mutations with potentially negative effects. As such, cells have evolved extensive mechanisms to detect DNA DSBs, to stabilize the broken DNA sites, and to manipulate the nucleic acid in an effort to repair the DNA while ensuring normal function. Given this critical and complex task, it is not surprising that eukaryotes have evolved multiple DNA DSB repair pathways that are generally conserved across eukaryotic life (Friedberg 2003, Mehta and Haber 2014, Ceccaldi et al. 2016). At least two of the DNA DSB repair pathways in eukaryotes are also active in prokaryotes, reviewed previously (Aravind et al. 1999, Cromie et al. 2001, Wright et al. 2018), highlighting the critical need to resolve DNA DSBs to maintain normal genome function. As detailed in this review, DNA repair is not a one-size fits all mechanism, and variation exists for the preference, protein components, and hierarchy of DNA DSB repair pathways (Lieber 2010, Bertrand et al. 2019). This review does not cover programed DNA DSBs induction (Borde and de Massy 2013) needed for genome function, such as class-switch recombination important for antibody diversification (Stavnezer et al. 2008), gene-conversion such as mating type switching (Haber 2012), or the regulation of DSB repair pathways required for telomere maintenance (Doksani and de Lange 2014).

The aim of this review is to detail DNA DSB repair, and to explore how these pathways influence modern genome engineering efforts and genome evolution in natural populations. We focus on experimental knowledge from animal, plant, and yeast models, and compare this with experimental results from filamentous pathogens. We specifically identify the study species with respect to experimental results where possibly, but generally, our references to yeasts refer to conventional species, such as Baker's yeast, *Saccharomyces cerevisiae*, which has been a critical model for studying eukaryotic DNA repair mechanisms. In addition to filamentous fungi, dimorphic fungi such as the model plant pathogens *Ustilago maydis* and *Zymoseptoria tritici*, along with oomycetes are discussed as well. The review is organized in four sections: (i) The basic molecular mechanisms of the four major DNA DSB repair pathways, including canonical non-homologous end joining (C-NHEJ), homologous recombination (HR), microhomology-mediated end joining (MMEJ) and single-strand annealing (SSA). We pay special attention to the MMEJ pathway, and the variety of other alternative end-joining pathways that have been described, as many details, function, and impact of these pathways remain unclear. (ii) Knowledge about the hierarchy of DNA repair pathways, and the factors that influence the interplay between pathway activity. (iii) How DNA repair influences genome engineering outcomes. (iv) The impact of DNA repair on genome evolution. We pay special attention to filamentous pathogens in section four, as previous observations on genome variation and evolution have detailed biased genome evolution, and we suggest this may be driven in part by variation in DNA repair, which could influence the emergence of pathogen genome variation.

## Overview of eukaryotic DNA double-strand break repair pathways in model systems, and knowledge gaps in filamentous pathogens

### Repairing DNA through canonical non-homologous end joining and homologous recombination

**Figure 1.**
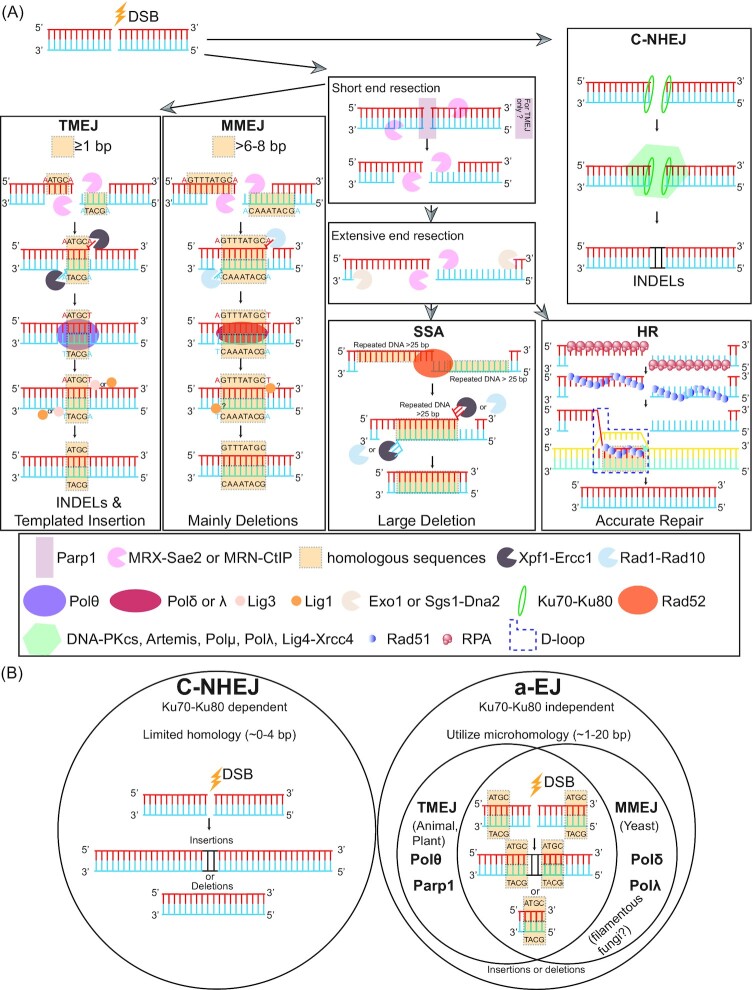
Schematic overview of the four DNA double-strand break repair pathways and diagram on the terminology for a-EJ, MMEJ, and TMEJ. **(A)** Following the formation of a DNA double strand break (DSB), two routes of repair can occur. If end resection proceeds, one of TMEJ, MMEJ, SSA or HR will predominately repair the DSB, while C-NHEJ is dominant in the absence of end resection. TMEJ or MMEJ proceed after short end resection mediated by MRX-Sae2 (budding yeast) or MRN-CtIP (mammals) that expose external microhomologous (MH) sequences at the DSB ends. Complementary base pairing between MH sequence directs repair. Further 3′ flap removal, gap-filling and ligation steps are required for proper TMEJ or MMEJ, while the protein components involved in these steps vary between TMEJ and MMEJ (e.g. PolΘ vs Pol δ- λ in gap filling and Lig3 vs potentially Lig1 in ligation). The mutational outcome of TMEJ is INDELS and templated insertions, while deletions are more common following MMEJ. For SSA, extensive end resection mediated by Exo1 or Sgs1-Dna2 results in large homologous sequence sites (>25 bp) at the DSB ends, and annealing between homologous sequence is stimulated by Rad52. The 3′ flap trimming by Xpf1-Ercc1(mammals) or Rad1-Rad10 (budding yeast) causes large deletions between the homologous sequences, which is a commonly found following SSA repair. For HR, ssDNA overhangs is bound by RPA then replaced with Rad51. Rad51 promotes strand invasion and forms D-loop between homologous sequences and typically results in accurate repair. For C-NHEJ, the Ku70-Ku80 dimer protects the DSB ends from end resection and recruit other key components (including DNA-PKcs, Artemis, Pol μ, Pol λ, Lig4-Xrcc4) for repair. Repair following C-NHEJ often results in INDELs. **(B)** C-NHEJ is defined as Ku70-Ku80 dependent DSB repair pathway, which utilizes minimal homologous sequences for repair and commonly results in INDEL mutations. The term a-EJ is defined as an umbrella term for Ku70-Ku80 independent repair that often utilizes microhomologous sequence for DSB repair. The a-EJ term can be further defined into TMEJ in mammals and plants, involving PolΘ and PARP1, or MMEJ in yeast and presumably filamentous fungi, which might involve Pol δ and Pol λ. DSB repair by a-EJ should result in a DNA mutation owing to the initial end resection step and later 3′ flap removal.

Proper repair of DNA double-strand breaks (DSBs), caused by inherent mechanisms or external factors, are vital to the maintenance and function of the genome (Mehta and Haber 2014, Srinivas et al. 2019, Vitor et al. 2020). The association between aberrant DNA DSB repair and genetic disease has been reported in many systems (Aparicio et al. 2014, Helleday et al. 2014). The two most well characterized DNA DSB repair pathways are canonical non-homologous end joining (C-NHEJ) and homologous recombination (HR), also called homology direct repair (HDR). The mechanisms of repair for these two pathways are quite different, and largely determined by the initial fate of the DNA ends at the break site. During C-NHEJ, the Ku70-Ku80 heterodimer binds to the DNA DSB ends, protecting them from end resection and recruits other enzymes central to C-NHEJ repair (Mimitou and Symington 2010). This includes a DNA-dependent protein kinase catalytic subunit (DNA-PKcs), endonuclease Artemis, and DNA polymerase μ and λ to promote DNA end processing suitable for subsequent ligation. The DNA ligase IV (Lig4)-XRCC4 complex is required for this final ligation to seal the DNA DSB (Chang et al. 2017) (Fig. 1A). As the name suggests, the C-NHEJ process does not rely on homologous DNA templates for repair, however, short microhomology (up to 4 base pair) has been found at repair junctions during C-NHEJ (Daley and Wilson 2005, Pannunzio et al. 2018). DNA DSB repair from the C-NHEJ pathway often results in small sequences changes at the repair site. This is due to the action of nucleases such as Artemis and the associated DNA polymerases, which process and prepare the broken DNA ends for ligation. This error-prone repair is characterized by small insertions and deletions (INDEL), and tandem duplications (Schimmel et al. 2017, Her and Bunting 2018). It is important to note that while C-NHEJ is often described as inducing INDELS, and therefore as error-prone, precise C-NHEJ ligation has also been reported (Betermier et al. 2014, He et al. 2016). Such high-fidelity DNA repair is often unreported or intractable with common selection and sequencing-based DNA DSB repair assays, and may occur more frequently than currently appreciated.

Unlike C-NHEJ, which suppresses end resection, the HR pathways utilizes end resection to generate single-stranded DNA ends compatible for homologous recombination. During the initial steps of HR, the Mre11 endonuclease cleaves 5′-terminated DNA as part of the Mre11-Rad50-Xrs2 complex that is termed MRX in budding yeast *Saccharomyces cerevisiae*, while the homologous complex of Mre11-Rad50-Nbs1 is termed MRN in mammalian systems (Wright et al. 2018). The endonuclease activity of the MRX/MRN complex is further promoted by the nuclease termed Sae2 in *S. cerevisiae*, or CtIP in animals (Cannavo and Cejka 2014, Anand et al. 2016). An interesting feature of the MRX complex is that it can process DNA bidirectionally. The nick caused by MRX and Sae2 serves as the entry point for 3′-5′ exonuclease activity by Mre11, and 5′-3′ exonuclease activity by Exo1 and/or helicase Sgs1 with endonuclease Dna2 (Garcia et al. 2011, Symington 2014). The result of DNA end resection by MRX and accompanying nucleases are long 3′ ssDNA overhangs that are bound by replication protein A (RPA), and subsequently replaced by DNA recombinase protein Rad51 with the help from accessory protein (i.e. Rad52 in budding yeast) (Chen et al. 2013, Daley et al. 2014). The association of Rad51 on ssDNA creates nucleoprotein filaments that promote base-pairing between ssDNA and homologous dsDNA in the genome. This process is referred to as strand invasion, and forms a DNA structure called a displacement loop (D-loop) (Wright et al. 2018) (Fig. 1A). The complimentary base pairing by the 3′ DNA sequence from the DSB site can serve as a DNA polymerase initiation site, allowing for extension of the invading strand with the homologous locus serving as a template (Wright et al. 2018). There are different mechanisms to resolve the DNA DSB depending on the D-loop disassociation, engagement of a second resected end, and the method resolving the double Holliday junction (dHJ) (Daley et al. 2014). The different HR outcomes can be achieved by resolving the extend D-loop by synthesis-dependent strand annealing (SDSA) pathway (the invaded strand with extended sequences gets dismantled for the D-loop and anneals with ssDNA from the other side of DSB); double-strand break repair (DSBR) pathway (second strand capture with dHJ resolution); double Holliday Junction dissolution pathway (second strand capture with dHJ dissolution) and break-induced replication (BIR) pathway (only one side of DSB gets repaired) (Daley et al. 2014, Kramara et al. 2018). In general, DNA DSBs repaired with HR result in a more precise sequence repair compared to C-NHEJ, owing to the use of homologous DNA template from either sister chromatids or homologous chromosomes (Wright et al. 2018). This fact serves as the basis for knock-in or gene insertion genome engineering projects that utilize extensive homologous sequence to stimulate site-specific insertion of exogenous DNA.

### Repairing DNA through microhomology-mediated end joining and single-strand annealing

There are additional DNA DSB repair pathways active in eukaryotes in addition to C-NHEJ and HR. These pathways are termed microhomology-mediated end joining (MMEJ) and single-strand annealing (SSA). Both pathways involve homologous DNA sequence to resolve the DNA DSB, but the pathways are different from each other, and from HR directed repair, based on the proteins involved, characteristics of the homologous DNA used for repair, and their resulting sequence profiles.

The generalized model for MMEJ across kingdoms of life involves end resection, annealing of microhomologous sequences from the exposed ssDNA ends, nuclease activity to remove DNA-flaps if necessary, DNA polymerase activity to fill gaps around the microhomology annealed sequences, and finally DNA ligation to seal the DNA DSB (Fig. 1A). The role of microhomologous sequence pairing during DSB end joining was first demonstrated in monkey cell lines (Roth and Wilson 1986). Genetic evidence of a Ku-independent repair pathway was first clearly demonstrated in *S. cerevisiae* using a Δ*ku70* strain that could *in vivo* repair a plasmid based DNA DSB that resulted in sequence deletion at the repair site (Boulton and Jackson 1996b). The size of the observed deletions ranged from 6 to 811 bp and were flanked by 3 to 16 bp microhomology. This work demonstrated the activity of two HR-independent repair pathways, and found Ku-dependent repair was preferentially active and less error-prone than Ku-independent repair (Boulton and Jackson 1996b). Another early study in *S. cerevisiae* using repair of non-complementary end sequences, also revealed that Ku-independent end joining resulted in sequence deletions and involved annealing microhomology sequences near the repair junction (Ma et al. 2003). This pathway was termed microhomology-mediated end joining (MMEJ) owing to the usage of microhomology to direct DNA repair (Ma et al. 2003). The terms alternative-NHEJ (a-NHEJ) and alternative end joining (a-EJ) have also been used to describe these DNA DSB repair outcomes that do not require known components of C-NHEJ (Fattah et al. 2010, Sallmyr and Tomkinson 2018). The occurrence of a-EJ repair mediated by microhomologous sequence has been readily observed in animal, plant, and fungal systems, suggesting an evolutionarily conserved outcome, however, the molecular mechanisms and complexes that mediate a-EJ across eukaryotic domains of life appear different (Fig. 1B). Work in multiple metazoan models, including mouse, *Drosophila melanogaster* and *Caenorhabditis elegans*, discovered that DNA polymerase theta (Polϴ) plays a key role in mediating a-EJ, characterized as C-NHEJ independent repair that utilizes short homologous sequence and often creates templated insertions (Shima et al. 2003, Chan et al. 2010, Roerink et al. 2014, Schimmel et al. 2019). The term theta-mediated end joining (TMEJ) has been proposed as a more precise definition for this repair, which falls under the broader umbrella term a-EJ that might include additional unknown repair mechanisms (Chan et al. 2010, Roerink et al. 2014) (Fig. 1B). In plants, there are clear homologs to animal Polϴ, and experimental evidence in the moss *Physcomitrella patens* suggests that the majority of DNA mutations following repair of Cas9-induced DSB are the result of Polϴ-mediated repair, independent of C-NHEJ (Mara et al. 2019). Many details regarding TMEJ repair appear similar between plants and animals (e.g. observed microhomology in the repair junctions) (van Kregten et al. 2016, Mara et al. 2019). Interestingly, the two most well studied phyla of fungi, namely *Basidiomycota* and *Ascomycota*, do not possess a clear homolog containing the canonical Polϴ domains (Meyer et al. 2015, Huang et al. 2021). Therefore, we will refer to DNA DSB repair results in fungi that involved microhomology and were C-NHEJ independent as MMEJ, while we will use the term TMEJ for similar DNA repair outcomes that involve Polϴ from animal systems.

Similar to the HR pathway, end resection is a key initial step for TMEJ to generate short ssDNA ends that may contain microhomology to direct annealing in animals. This initial end resection is carried out by the MRN complex, together with CtIP, as described for HR (Zhang and Jasin 2011, Truong et al. 2013, Ahrabi et al. 2016). This initial end resection by MRN results in relatively short DNA sequence removal prior to TMEJ. For HR, further end resection is carried out to expose longer ssDNA mediated by Exo1 or BLM-Dna2, which are generally dispensable for TMEJ (Truong et al. 2013). Another early molecular event implicated in TMEJ is PARylation (i.e. post-translational deposition of ADP-ribose molecules) of proteins associated with the DSB site by poly (ADP-ribose) polymerase 1 (PARP1) (Ray Chaudhuri and Nussenzweig 2017). The model is that PARylation serves as a signal to recruit additional proteins (e.g. Mre11) to the DSB site to facilitate TMEJ-mediated repair, and genetic and chemical inhibition of PARP1 activity reduces TMEJ repair (Mansour et al. 2010, Wray et al. 2013, Ray Chaudhuri and Nussenzweig 2017). Following unpaired DNA end trimming by Xpf-Ercc1 (Bai et al. 2021), the 3′OH group is eligible for polymerase-mediated DNA synthesis (Sijbers et al. 1996). The major factor defining TMEJ in animals and plants is the function of a low-fidelity DNA polymerase theta (PolΘ) encoded by *POLQ* (Kent et al. 2015, Mara et al. 2019). PolΘ homolog (mus308) was first identified from a screening assay with hypersensitivity to DNA crossing-linking agents in *Drosophila* (Boyd et al. 1981, Boyd et al. 1990). Further molecular data suggested that PolΘ is able to extend the single-strand DNA with either template-independent or -dependent activities (Hogg et al. 2012, Kent et al. 2015, Kent et al. 2016). The final step for DSB repair is to seal the nick between the two ends through a phosphodiester bond, catalyzed by two DNA ligases in animals, termed ligase I (Lig1) and ligase III (Lig3) (Liang et al. 2008) (Fig. 1A).

There is comparatively less mechanistic evidence for Ku70-80 independent DSB repair pathway in fungi, referred to here as MMEJ. The majority of work published to date has been conducted in yeast, an incredibly productive system, but hardly representative of all fungi. The initial end resection during MMEJ is carried out by the same complex as in HR, MRX with Sae2 (Sfeir and Symington 2015). Conflicting results have been reported in budding yeast regarding the role of the MRX complex and MMEJ. Using a chromosomal end joint assay, one group reported that Δ*sae2* and Δ*mre11* mutants without nuclease activity appeared to have normal MMEJ repair (Deng et al. 2014), while earlier observations using a dual endonuclease HO-mediated cleavage assay reported that Δ*sae2* and Δ*mre11* caused MMEJ defects (Lee and Lee 2007). The difference may be due to the distinct experimental conditions or point to unknown differences in end resection requirements in this yeast. In animals, experimental evidence links PARylation by PARP1 to TMEJ repair, but the role of PARylation during fungal DNA repair remains unclear (Audebert et al. 2004, Tao et al. 2009, Citarelli et al. 2010, Ray Chaudhuri and Nussenzweig 2017). In *Aspergillus nidulans*, a putative PARP homolog, termed PrpA, was identified that contained high identity to the catalytic domain of PARP1, but lacked the N-terminal DNA binding domain (Semighini et al. 2006). Assays to test for altered sensitivity to DNA damaging agents, protein localization and transcriptional responses indicated that PrpA may play a role in DNA damage, but direct evidence was not provided (Semighini et al. 2006). The PrpaA homolog in *Neurospora crassa*, termed NPO, was shown to be a *bona fide* PAR-polymerase, and the gene was transcriptionally induced by the DNA damage agent methyl methanesulfonate (MMS), but unlike in *A. nidulans*, the Δ*npo* strain in *N. crassa* was not sensitive to DNA damaging agents (Kothe et al. 2010). The observations that PrpaA was required for normal growth and development in *A. nidulans*, but was dispensable in *N. crassa* indicates divergent function, and further research is needed regarding the role of PARP homologs for fungal DNA damage response. Following end resection, ssDNA with homologous sequence anneals, and 3′ flap DNA is excised by the complex of Rad1 and Rad10, endonucleases discovered in *S. cerevisiae* (Ma et al. 2003). The absence of a clear PolΘ homolog, containing the characteristic domains, is the major difference suggesting altered mechanisms for DNA DSB repair utilizing microhomology in animals and fungi (Wood and Doublie 2016, Huang et al. 2021). In *S. cerevisiae*, there is evidence that DNA polymerases 3 (Pol δ) and DNA polymerase 4 (Pol λ) function in gap-filling (i.e. DNA synthesis) following microhomology annealing (Meyer et al. 2015). Why there are two polymerases, how they interact, and their overall function in MMEJ in filamentous fungi remains to be determined (Meyer et al. 2015). For the final DNA ligation step, the main ligase from animals, Lig3, is absent in *S. cerevisiae*, and while a Lig1 homolog exists, its role in MMEJ has not been tested (Audebert et al. 2004, Liang et al. 2008, Sfeir and Symington 2015). In addition to the molecular differences described between TMEJ in animals and MMEJ in fungi, the sequence requirements and outcomes also appear to vary. In *C. elegans*, only ∼1 bp of microhomology appears to be required to initiate TMEJ, and 2–4 bp of microhomology is frequently observed in *Xenopus* (Koole et al. 2014, van Schendel et al. 2015, Chandramouly et al. 2021). However, in two separate studies in *S. cerevisiae*, longer stretches of microhomology between 8 and 20 bp were observed (Villarreal et al. 2012, Lee et al. 2019). Following TMEJ repair in metazoans, small insertions/deletions (INDELS) and templated insertions are predominant at the DSB repair sites, while insertions are rare in yeast (Sfeir and Symington 2015, Ceccaldi et al. 2016, Schimmel et al. 2019) (Fig. 1A).

Single-strand annealing (SSA) initiates through the same end resection step mediated by the MRN complex to generate free 3′-ssDNA for homology search, a shared requirement for SSA and TMEJ/MMEJ (Bhargava et al. 2016). However, one of the main differences between SSA and TMEJ/MMEJ is the length requirement between annealed homologous sequence (Sallmyr and Tomkinson 2018). Estimates vary, but SSA is generally characterized to require homology > 25 bp, which is longer than what is generally thought to be used for TMEJ/MMEJ (Sallmyr and Tomkinson 2018). The use of longer homologous sequence is likely the results of a second round of end resection mediated by Exo1 or Sgs1-Dna2 (Sturzenegger et al. 2014). This more extensive end resection generates longer ssDNA filaments that are bound by RPA and can undergo homology directed annealing, stimulated by RAD52 and small acidic protein DSS1 (Grimme et al. 2010, Stefanovie et al. 2020). The 3′ unpaired tail DNA is removed by Xpf1-Ercc1 during both SSA and TMEJ/MMEJ repair (Al-Minawi et al. 2008). The final steps include gap-filling by DNA polymerase and ligation by DNA ligase, but the exact protein requirements for these steps are poorly defined (Bhargava et al. 2016). The result of SSA repair is often large deletions flanked by homologous sequence, which are frequently genomic repeats, that result from homologous pairing of distantly located loci followed by non-homologous 3′ flap trimming before ligation (Bhargava et al. 2016) (Fig. 1A).

### Interactions between DNA DSB repair pathways and knowledge gaps in filamentous pathogens

Given the function of multiple DNA repair pathways with a similar purpose, it is no surprise that multiple studies have detailed molecular interactions between the pathways. Evidence suggests the interactions depend on multiple cellular, genomic, and physiological factors that are discussed in section two of this review. Here we discuss general observations and molecular interactions between the repair pathways that influence the hierarchy of DNA repair. Generally, C-NHEJ is the predominant pathway to resolve DNA DSBs in multiple eukaryotic organisms, including mammalian cells, somatic plant cells, and filamentous fungi (Ninomiya et al. 2004, Knoll et al. 2014, Chang et al. 2017). The binding of the Ku70-Ku80 heterodimer to DSB ends represses end resection that is required for HR, TMEJ/MMEJ and SSA (Shao et al. 2012). The general evidence for this is that other repair pathways are generally difficult to detect, but are easily observed when core C-NHEJ components (e.g. Ku70-Ku80) are deleted (Boulton and Jackson 1996b, Ninomiya et al. 2004, Chu et al. 2015). Following MRX and Sae2-mediated short end resection, Ku70-Ku80 DNA binding is inhibited and TMEJ/MMEJ repair are more prominent (Lee and Lee 2007, Ceccaldi et al. 2016). This is seen in *S. cerevisiae*, where removal of Sae2 or expression of a nuclease defective Mre11 variant results in higher repair activity of C-NHEJ (Lee and Lee 2007). Similarly, the amount of Ku protein recruited to DSB sites was increased in a Δ*mre11* mutant (Zhang et al. 2007). For other pathway interactions, removal of PolΘ in mammalian cells leads to increased HR repair (Mateos-Gomez et al. 2015). If extensive end resection is initiated, several hundred or thousands of DNA bases can be removed, and the SSA and HR pathways become predominant (Ceccaldi et al. 2016). Following long DNA end resection, the TMEJ/MMEJ pathway is inhibited by RPA binding ssDNA ends (Deng et al. 2014). Disrupting the HR pathway, such as through Δ*rad51* deletion, promotes the frequency of MMEJ or SSA (Mansour et al. 2008, Deng et al. 2014). Due to the overall similarity between TMEJ/MMEJ and SSA, the molecular interactions and hierarchy between them is less clear. Collectively, evidence from genetic and biochemical experiments show that there is competition and inhibition between repair pathways for a given DNA DSB.

The majority of data on DNA repair in fungi come from studies on a few species. Early work in fungi on DNA repair and recombination were critical to the development of modern theory, relying heavily on experimental results from *S. cerevisiae, N. crassa*, and the *Basidiomycete Ustilago maydis*, the causal agent of corn smut (Inoue and Schroeder 1988, Stahl 1994, Boulton and Jackson 1996a, Kojic et al. 2003, Ninomiya et al. 2004, Steinberg and Perez-Martin 2008, de Sena-Tomas et al. 2015). However, this represents a small sampling of fungal diversity, and the early research does not incorporate current knowledge from metazoan models to understand if the rules and hierarchy discovered in those systems pertain to filamentous microbes. For instance, it is not clear how exciting new research defining the role of PolΘ influencing genome stability and cancer development, pertain to fungi given that the major phyla lack a clear homolog of PolΘ (Brambati et al. 2020, Huang et al. 2021). As the molecular mechanisms, outcomes, and importance of MMEJ are not well defined across diverse fungi, it is difficult to compare and contrast results between metazoans and fungi. It is also important to note that some DNA repair details in *S. cerevisiae*, such as the predominant action of HR, do not appear to reflect the majority of filamentous fungi that exhibit dominant repair by C-NHEJ (Haber 1999, Ninomiya et al. 2004). Thus, caution is warranted when extrapolating details across fungal taxa. More research is needed across filamentous fungi and oomycetes to molecularly characterize and document that action of DNA DSB repair mechanisms, interactions, and how this critical cellular process influences stability and evolution of fungal genomes.

## Factors affecting the usage of DNA repair pathways and their conservation in filamentous pathogens

### Multiple repair pathways for a single DNA DSB-who repairs what?

Given the potentially lethality of unresolved DNA DSBs, it is not a surprise evolution would give rise to multiple DNA repair pathways in the cell. However, the occurrence of partially overlapping and redundant pathways to achieve the same function raises the question of which pathway should repair any given DNA DSB? In the following section, we summarize current knowledge for how cell physiology and genomic features influence DNA DSB repair pathway choice.

### Cell cycle

**Figure 2.**
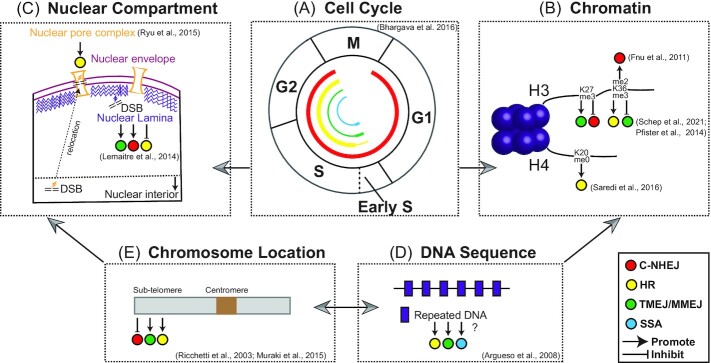
Overview of how physical, chemical, and biological conditions influence the usage of different DNA repair mechanisms. **(A)** The choice of DNA repair is tightly controlled by cell cycle. C-NHEJ is activated throughout cell cycles but dominates in G1 phase. TMEJ/MMEJ, SSA and HR are mainly functional from S to G2 phase, while HR becomes more dominant after early S phase when sister chromatids are available as repair templates (Bhargava et al. 2016). The thickness of the lines indicates the activity of different DNA repair pathway in the cell cycle. **(B)** Local chromatin status affects the choice of DNA repair pathway. For example, TMEJ has been found toward H3K27me3 involved heterochromatin from Schep et al. 2021. The positive or negative roles of H3K36me2/me3 in regulating C-NHEJ, HR or TMEJ has also been reported in multiple studies (Fnu et al. 2011 and Pfister et al. 2014). Besides, uniquely unmethylated lysine residues H4K20me0 on new histones deposited during DNA replication promotes HR repair (Saredi et al. 2016). **(C)** Nuclear compartments and nuclear positioning are associated with DNA repair patterns. For example, to avoid ectopic recombination, DSB inside heterochromatin relocates to nuclear periphery to induce the error-free HR repair (Ryu et al. 2015), while heterochromatic DSB arising from nuclear lamina remains positionally stable, promotes TMEJ and C-NHEJ and impairs HR (Lemaitre et al. 2014). **(D and E)** Sequence content and physical chromosome location have influence on DSB repairs. The influence of repeated DNA on HR-mediated genome variation has also been reported (Argueso et al.^2008^). We propose that repeated DNA might promote to HR, TMEJ/MMEJ and SSA due to the usage of homologous DNA in these repairs. Additionally, it has been found that sub-telomere DSB suffers more from TMEJ/MMEJ and HR than C-NHEJ (Ricchetti et al. 2003, Muraki et al. 2015). These factors do not function alone, but interact to influence DNA repair pathway choice, indicated by arrows between the panels.

DNA DSB repair is tightly controlled during the cell cycle (Ceccaldi et al. 2016, Chang et al. 2017) (Fig. 2A). C-NHEJ is activated throughout all phases (i.e. G1, S, G2, M) and dominates in G1. The mammalian protein, 53BP1 together with its downstream effector RIF1, inhibit end resection at DSB sites, limiting the recruitment of homology-dependent repair proteins to DSB sites in a cell cycle dependent manner. Inactivation of 53BP1 or RIF1 causes increased HR, MMEJ, and SSA repair frequencies (Escribano-Diaz et al. 2013). Experiments in human cell lines show that during the S and G2 phases, C-NHEJ is suppressed by cell cycle regulator of NHEJ (CYREN, also known as MRI) via interacting with Ku proteins (Arnoult et al. 2017). There is also a report that CYREN can promote C-NHEJ in the absence of the XRCC4-like factor, termed XLF in the G1 stage (Hung et al. 2018). The XLF protein is crucial during the final ligation reaction of C-NHEJ by interacting with Lig4-XRCC4 (Ahnesorg et al. 2006), and these observations indicate diverse control of DNA repair pathways in a cell-cycle dependent manner.

The HR pathway is more active during the S and G2 cell phases, when DNA replication has taken place and nucleic acids to serve as repair templates are available. The activity of cyclins and cyclin-dependent kinases (CDKs) promote DNA end resection and HR activity in a cell-growth dependent manner (Aylon et al. 2004, Mathiasen and Lisby 2014). In *S. cerevisiae*, Cdk1 (also known as Cdc28) phosphorylates Sae2 (Ser267) to facilitate short end resection in the S/G2 phase. A serine to alanine (S267A) substitution, creating a Sae2 phosphorylation deficient mutant, mimics DNA repair defects observed in a Δ*sae2* null mutant, while serine to glutamic acid (S267E) substitution (i.e. phosphomimic) phenocopies wild-type Sae2 in the absence of Cdk1 (Huertas et al. 2008). Further studies in *S. cerevisiae* suggest that phosphorylation causes tetramerization of Sae2, followed by activation of Mre11 and interaction with Rad50, which promotes MRX-mediated end resection (Cannavo et al. 2018). The resection nuclease, Dna2, involved in extensive end resection, is also regulated by Cdk1 phosphorylation (Chen et al. 2011). Additionally, multiple HR related proteins involved in end resection, DNA damage checkpoint or recombination have been also identified as Cdk substrates from yeast and mammalian systems (Zhao et al. 2017). A recent study in *S. cerevisiae* found that Rad51 and Rad52 are phosphorylated by Cdk1 during G2/M phase with related cyclins (e.g. Clb2 and Clb3), and phosphorylated Rad51 and Rad52 promote DNA binding and strand annealing, respectively (Lim et al. 2020). Given the role of Rad52 in mediating ssDNA annealing during SSA repair, it is possible that SSA activity is cell cycle dependent. It would be interesting to further test how cell cycle regulated Rad52 impacts DNA repair pathway activity between HR and SSA (Grimme et al. 2010, Bhargava et al. 2016, Lim et al. 2020). An important future question is the role, hierarchy, and interaction of various Cdk phosphorylation targets. Understanding this network will provide a clearer understanding of DNA repair pathway activity in the context of the cell cycle.

It is less clear how TMEJ/MMEJ and SSA repair pathways are impacted by the cell-cycle. There are conflicting reports on the role of 53BP1, which was shown to facilitate TMEJ during G1 in human cell lines (Xiong et al. 2015), but it was shown to suppress TMEJ, HR, and SSA repair under similar human cell line conditions (Munoz et al. 2012). The TMEJ/MMEJ pathway is presumably favored over SSA in G1 in the absence of C-NHEJ due to the shorter homologous sequence requirement (Chang et al. 2017). Previous models proposed that end resection dependent repair pathway preference is governed by the availability of repair templates. This model suggests that TMEJ/MMEJ and SSA are preferred to HR when sister chromatids are not available in early S phase, and then later following DNA synthesis, HR is the predominant DNA DSB repair pathway (Bhargava et al. 2016). To our knowledge, there is little experimental evidence regarding DNA repair choice and cell cycle dependence in filamentous pathogens.

Knowledge generated in animal and yeast systems may transfer to filamentous fungi, but it is reasonable to believe that significant differences may exist because of inherent biological differences between classes of eukaryotes. We ask the reader to keep in mind that while DNA repair is critical, and the process is generally conserved across prokaryotic and eukaryotic life, it should not be assumed that the mechanisms or the impact on the genome are the same (Steenwyk et al. 2019). Factors such as ploidy, where animal models are commonly diploid and many filamentous fungi are haploid, likely impacts DNA repair. Many animal systems, and some fungal systems (e.g. yeasts) frequently use the sexual cycle, but it is absent or not demonstrated in many filamentous pathogens. Also, some filamentous fungi are multi-nucleate, which can impact genome evolution (Roper et al. 2011), but details regarding how multiple nuclei in a cell could impact the DNA repair process remains unknown.

### Chromatin status and nuclear compartmentalization

Local chromatin (i.e. DNA-RNA-protein interactions) can influence DNA DSB repair outcomes (Clouaire and Legube 2019, Scully et al. 2019) (Fig. 2B). A majority of studies to-date have focused on how chromatin impacts C-NHEJ versus HR, but this could be caused more by experimental bias than the importance of chromatin influencing all types of DNA DSB repair pathway preference. Methylation at histone 3 lysine 36 (H3K36me) has been extensively studied in animals and yeast for its role in DNA repair (Sun et al. 2020). For example, di-methylation at histone 3 lysine 36 (H3K36me2) catalyzed by a SET-domain containing methyltransferase (i.e. Metnase), localizes to induced DSB sites and promotes C-NHEJ repair through recruitment of Ku70 in human cell line experiments (Fnu et al. 2011). Post-translational modification of H3K36 appears to also influence DNA repair in fission yeast *Schizosaccharomyces pombe*, where data show antagonism between Set2-mediated H3K36 methylation promoting C-NHEJ, and Gcn5-mediated H3K36 acetylation promoting HR (Pai et al. 2014). However, in human cell lines, Setd2-dependent H3K36me3 can recruit the nuclease CtIP, through the H3K36me3 reader protein LEDGF, to promote end resection and HR (Pfister et al. 2014). Biological variation, such as differences in histone modifications or their reader proteins (i.e. epigenome), may influence organism specific DSB preference, such as seen between humans and yeast (Pai et al. 2014, Pfister et al. 2014). For instance, the difference for H3K36me3 being associated with C-NHEJ in yeast, but with HR in humans, may be attributed to yeast lacking a LEDGF homolog. In addition to H3K36 modifications, other histone modifications, such as H3K4me2, H3K9me3, H3K79me2, H4K16ac and H4K20me0/2, can affect DSB repair choice (Alagoz et al. 2015, Saredi et al. 2016, Pellegrino et al. 2017, Clouaire et al. 2018, Clouaire and Legube 2019, Horikoshi et al. 2019). For example, structural and biochemical studies found that new histones deposited during DNA replication have uniquely unmethylated lysine residues (H4K20me0), while old histones are mainly methylated (H4K20me1/2/3) (Saredi et al. 2016). The post-replicative chromatin accompanying H4K20me0 can promote HR repair through interaction with the HR related protein complex TONSL-MMS22L. However, mono- or di- methylation at H4K20 are not compatible with TONSL binding (Saredi et al. 2016). Additionally, dilution of H4K20me2 after replication affects 53BP1 binding, which helps limit end resection, and thus promotes HR repair (Pellegrino et al. 2017). Moreover, genome-wide mapping of histone modifications via ChIP-seq in human cell lines revealed the association between the enhanced levels of multiple histone modifications (e.g. H3K36me3, H3K79me2 and H3K4me2) and potential HR repaired regions. These transcription activation markers might provide a preferred chromatin status for HR machinery (Clouaire et al. 2018, Her and Bunting 2018). This is consistent with other mechanistic data, such as the bromodomain containing protein, ZMYND8 identified in human cell lines, that interacts with acetylated histones at transcriptionally active chromatin, and recruits the nucleosome remodeling and histone deacetylase (NuRD) complex to promote HR repair (Gong et al. 2015). In *Arabidopsis*, a cohesion accessory protein, PDS5C (also known as RDM15), interacts with H3K4me1 at actively transcribed chromatin, and the complex is important for HR DSB repair (Pradillo et al. 2015, Niu et al. 2021, Quiroz et al. 2022). In addition to euchromatin, transient formation of H3K9me3-mediated heterochromatin can accumulate at DNA DSB sites, which is thought to stabilize the damaged chromatin and activate further repair via regulating the level of acetyltransferase Tip60 and ATM kinase (Ayrapetov et al. 2014). Collectively, these observations show that multiple chromatin factors related to accessibility and transcriptional status influence the choice between C-NHEJ and HR repair (Aymard et al. 2014, Jha and Strahl 2014, Pai et al. 2014).

Compared to chromatin effects on C-NHEJ and HR, much less is known regarding the influence on TMEJ/MMEJ and SSA. A short reporter sequence inserted at over 1000 genomic loci in human cell was used to track DNA repair following Cas9 induced DSBs, and found that C-NHEJ is generally biased toward euchromatin, while TMEJ is more probable in H3K27me3 associated heterochromatin. Inhibition of H3K27me3 methyltransferase EZH2, a component of the PRC2 complex, shifts the repair preference toward C-NHEJ for previously TMEJ preferred sites (Schep et al. 2021). A separate study showed that chromatin decompaction followed by hypotonic stress leads to enhanced SSA and repressed HR in DSB repair (Krieger et al. 2021).

In addition to chemical modifications at histone residues, the physical nuclear location of DNA (i.e. nuclear compartments) appears to affect DNA DSB repair choice (Fig. 2C). Nuclear compartments are membraneless boundaries that have been documented based on morphological and functional studies, and include multiple regions occurring in the interior, such as the nucleolus, nuclear speckles, Cajal bodies, PML bodies, and regions on the nuclear periphery, such as nuclear lamina, nuclear envelope, and nuclear pores (Belmont 2021). In *S. cerevisiae*, DSB sites occurring on DNA located in internal nuclear regions can migrate to nuclear pore complexes for repair (Nagai et al. 2008). Dual mutations of Nup84, a nuclear pore complex, and Rad27, a HR related protein, results in a synthetic lethal phenotype (Loeillet et al. 2005). These observations suggest that nuclear compartments and nuclear positioning contribute to DNA repair (Loeillet et al. 2005, Nagai et al. 2008, Lamm et al. 2021). Concordantly, a pioneering study in *Drosophila* found that DSBs in heterochromatic DNA relocate to the nuclear periphery, mediated by SUMOylation and other protein components. This relocation avoids ectopic recombination at heterochromatic DSBs composed largely of repeated sequences, and promotes higher fidelity HR repair (Ryu et al. 2015). Interestingly, taking advantage of human cell lines, Lemaître and colleagues found that DSBs occurring in DNA at the nuclear lamina did not relocate and remained positionally stable. This result reported that repair of DSBs at nuclear lamina with compact heterochromatin were impaired in HR while C-NHEJ and TMEJ were activated (Lemaitre et al. 2014). A distinct DNA repair pattern has also been reported to occur for centromeric and pericentric heterochromatin in mouse cells, indicating that repair of pericentric heterochromatin favors C-NHEJ repair within the nuclear core in the G1 phase, and relocates to the nuclear periphery for HR repair in S/G2 phase, while DSBs arising at centromeric heterochromatin are located in the nuclear periphery and recruit C-NHEJ or HR factors in a cell cycle independent manner (Tsouroula et al. 2016). These data provide a clue for an additional layer of information and regulation of DNA repair in eukaryotes.

ChIP-seq and ATAC-seq enable genome-wide mapping of histone modifications and chromatin accessibility, and have been applied in multiple filamentous pathogens (Connolly et al. 2013, Moller et al. 2019, Cook et al. 2020, Fang et al. 2020, Zhang et al. 2021). Most work in filamentous pathogens has focuses on gene regulation or genome structure, not DNA repair. Interestingly, evidence from short-term lab evolution assays in the wheat pathogen *Z. tritici*, found that H3K27me3 is associated with certain unstable genomic regions and increased mutation rates, while H3K9me3 stabilizes the genome (Moller et al. 2019, Habig et al. 2021). Alterations in DNA repair mediated by a specific histone code is one hypotheses to explain these observations (Habig et al. 2021). Interestingly, many gene deletion assays using either traditional HR strategies or CRISPR-mediated editing have reported variation in gene deletion efficiency among the different loci in filamentous pathogens (Villalba et al. 2008, Ah-Fong et al. 2021). The influence of chromatin in determining these results offers an attractive hypothesis, but future studies are needed to understand the role of chromatin features in DSB repair choice and editing efficiency, especially in filamentous microbes.

### Sequence content and physical chromosome location

The underlying DNA sequence and physical location along the chromosome can influence the outcomes of DSB repair (Fig. 2D and E). For example, the resulting mutations from Cas9 editing and repair, namely insertions and deletions, can be precisely predicted based on the fourth nucleotide upstream of the PAM sequence based on experiments in human cells (Chakrabarti et al. 2019). In support of this, target flanking sequence dependent repair was found in a large-scale investigation of Cas9 induced DNA DSB, using more than 40 000 guide RNAs tested in multiple genetics backgrounds (Allen et al. 2018). The authors found that editing outcomes can be accurately predicted through local DNA sequence alone (Allen et al. 2018). Additionally, mismatch, GC-content and length within microhomologous sequence influences the efficiency of TMEJ/MMEJ, probably by affecting the stability of annealed intermediate (Daley and Wilson 2005, Villarreal et al. 2012, Kent et al. 2015, Meyer et al. 2015, Lee et al. 2019). It is reasonable to speculate that genomic regions rich in repetitive sequences, that can serve as homologous templates during HR, TMEJ/MMEJ, and SSA, might be involved in these types of repair more frequently than other regions lacking high proximal-homology. Consistently, HR repair at repetitive sequences has been identified as the source of genomic variation (Argueso et al. 2008). Similarly, we have observed frequent DNA deletions and insertions following CRISPR induced DSBs at a repeat rich locus in *Magnaporthe oryzae* (Huang et al. 2021).

Genome-wide targeting in mouse cells of ∼1000 integrated reporter genes in different locations revealed that genomic location contributed to variation in mutation frequency owing to unknown factors (Gisler et al. 2019). A series of studies in mammalian cells reveal that telomeric DSB results in more frequent large deletion and chromosome rearrangements owing to Mre11-mediated 3′ to 5′ exonuclease processing during HR and TMEJ accompanying with reduced C-NHEJ in telomere, compared to interstitial DSB. Interestingly, the reduced rate of C-NHEJ was also observed in interstitial DSBs with adjacent telomeric repeat sequences (Zschenker et al. 2009, Miller et al. 2011, Muraki et al. 2015). Investigation on yeast chromosome XI showed that the rate of C-NHEJ decreased from the internal chromosome to the telomere, while other repair pathways (e.g. microhomology mediated BIR) were involved in sub-telomeric repair (Ricchetti et al. 2003). Difference in DNA sequences might also contribute to DNA repair variation between centromeric and pericentric heterochromatin (Tsouroula et al. 2016). In filamentous pathogens, such repair preference may explain observations such as the variable stability of Avr proteins. For example, the avirulence gene *AvrPi9* and corresponding resistance gene *Pi9* from the rice blast pathosystem, has provided durable resistance in commercial rice production that may have to do with *AvrPi9* stability (Qu et al. 2006, Wu et al. 2015). Interestingly, this effector is located near the centromere in *M. oryzae*, and it is interesting to speculate that this chromosomal location impacts its variability in the population (Wu et al. 2015). While in the human pathogen *Cryptococcus neoformans*, C-NHEJ and HR both appear to be involved in repairing DSBs at repeated centromere sequences (Yadav et al. 2020). Additional efforts are needed for further understand the role of chromosome location in affecting the choice and outcome of DNA DSB repair in filamentous pathogens.

## The influence of DNA repair pathway usage on the full spectrum of CRISPR genome editing mutations

Following the first *in vivo* genome editing using CRISPR-Cas9 in human cell lines, CRISPR-Cas DNA editing systems have revolutionized genetics and functional genomics (Cong et al. 2013, Mali et al. 2013, Hsu et al. 2014). The majority of CRISPR-Cas systems used for genome engineering rely on single effector Type-II Cas proteins with intrinsic nuclease activity. Therefore, following the generation of Cas-mediated DNA DSBs, the endogenous DNA repair machinery is activated for repair. Recent reviews have detailed how DNA repair pathways affect genome editing outcomes in model systems (Xue and Greene 2021, Nambiar et al. 2022), but our knowledge remains limited for filamentous pathogens. More recent efforts for genome engineering have developed modified CRISPR platforms that do not create DSBs, such as PRIME-editing, CAST and Drag-and-drop approaches (Anzalone et al. 2019, Strecker et al. 2019, Ioannidi et al. 2021). The development of these modified systems is in part a response to observations that CRISPR-Cas genome engineering can create substantial unintended DNA modifications, likely owing to the creation of DNA DSBs. Such observations have focused on so-called off-target mutations, where a secondary locus to the CRISPR targeted locus has an induced mutation. However, CRISPR-Cas systems that create DSBs also frequently cause unexpected and aberrant outcomes at the primary, CRISPR targeted locus (Kosicki et al. 2018, Lee and Kim 2018). These on-target but unintended mutations are likely the results of the interplay of endogenous DNA repair pathways, unknown genomic rules, and physiological conditions during editing. In this section, we focus on reported low-fidelity and aberrant CRISPR on-target editing outcomes in model systems and filamentous pathogens and the potential link to DNA DSB repair pathway function.

### Genome editing in animals

**Figure 3.**
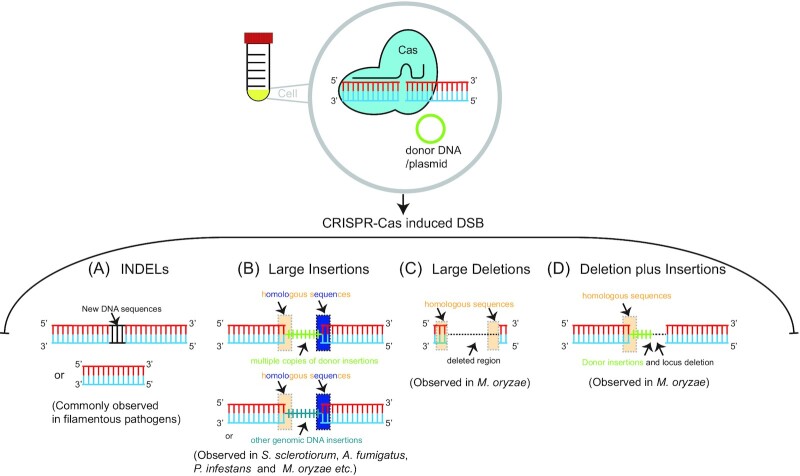
Aberrant DNA editing outcomes in filamentous pathogens. **(A)** INDELs are commonly observed Cas editing results in multiple organisms, including filamentous pathogens. Other types of DNA mutations following Cas-induced DNA repair, such as **(B)** large insertions, **(C)** large deletions, and **(D)** deletion plus insertions have been observed in multiple filamentous microbes.

In the absence of homologous DNA template for DSB repair, the majority of CRISPR-Cas endonuclease editing (e.g. Cas9 and Cas12a) results in small insertions or deletions (INDELs) at the target locus, thought to be mediated by C-NHEJ (Fig. 3A). The goal of most projects employing this approach is the creation of null-mutants, identified using amplification and sequencing based techniques to assess the target locus. Projects relying on amplicon sequencing have a limited ability to capture only certain DNA mutations at the target locus, as any mutation that alters primer binding or creates a sequence that is unamenable to amplification (e.g. large DNA insertion) will not be identified. This creates ascertainment bias and such analysis underestimates large-scale, complicated, error-prone DNA repair outcomes. Research specifically interested in identifying the broader class of DNA mutations following Cas-based editing have employed third generation long fragment DNA sequencing and more precise analysis, allowing for the characterization of previously hidden DNA mutations (Adikusuma et al. 2018, Kosicki et al. 2018, Cullot et al. 2019, Skryabin et al. 2020, Alanis-Lobato et al. 2021). For example, using PacBio long-fragment DNA sequencing, several classes of complex genome mutations were characterized at the primary on-target Cas9 editing locus in a mouse cell line. This systematic analysis of individual cell clones revealed that large deletions (up to 9.5 kb), large insertions (up to 2.5 kb), deletions plus large insertions, inversions, and INDEL mutations occurred at the on-target locus (Kosicki et al. 2018). Such mutations would have been missed with simple amplicon-based analysis (Kosicki et al. 2018). In another study using microinjected mouse embryos, frequent large DNA deletions, ranging from 100 bp to 3.2 kb were found at multiple Cas9 targeted loci. In addition, approximately 23% of sequencing reads suggested that exogenously supplied Cas9 and gRNA plasmid DNA were inserted at the target locus (Adikusuma et al. 2018). Similarly, unexpected segmental gains and losses near the Cas9 editing locus, and even the loss of an entire chromosome, were observed in experiments editing human embryos (Zuccaro et al. 2020, Alanis-Lobato et al. 2021). A key observation resulting from Cas9-mediated large deletions in mouse cell lines is the significant overrepresentation of microhomology (2 to 5 bp) observed at the break junction sites (Owens et al. 2019). This led to the hypothesis that TMEJ is responsible for creating the observed large deletions, but direct experimental evidence needed to rule out the involvement of other pathways was not provided (Owens et al. 2019). A recent approach more directly determines the involvement of specific repair pathways mediating Cas9 induced large deletions in mice cell lines by conducting editing in lines lacking 32 DNA repair genes (Kosicki et al. 2022). The authors reported an increased frequency of Cas9 mediated large deletions when editing in mice knock-out lines lacking major C-NHEJ components, including Ku70-Ku80, Lig4, and Xrcc4, while there was not an increase in large deletion outcomes for other minor C-NHEJ factors (e.g. Artemis). In addition, a reduced frequency of large deletions was detected in knock-out lines for genes involved in end resection and TMEJ, such as Nbs1 (yeast Xrs2 homolog) and the core TMEJ polymerase PolΘ (Kosicki et al. 2022). A new analysis pipeline, termed PEM-Q, found both an increased occurrence of large deletions and large DNA insertions (i.e. >20 bp) following Cas9-mediated editing in mouse cell lines with mutated Ku80 or Lig4 (Liu et al. 2021). Collectively, these results demonstrate frequent on-target mutations during genome editing in animal systems that are more complex than small INDELs and cause substantial DNA sequence changes. There is ample evidence of the underlying competition between DNA DSB repair pathways, especially between C-NHEJ and TMEJ, where the profile of the observed mutation outcomes changes when one pathway is inhibited. Interestingly, in mouse embryonic stem cells, Cas9 mediated INDELs created through C-NHEJ repair caused tandem duplications (e.g. short insertions creating repeat ‘ATT/ATT’), while TMEJ mediated repair mainly contributed to templated insertions (e.g. inserted sequence that is not contiguous, but in proximity ‘ATT(X)ATT’) (Schimmel et al. 2017). These results suggests that in addition to molecular competition between the pathways, there is functional variation for the resulting mutation profile dependent on the DNA repair pathways employed by the cell.

### Genome editing in microbes

While the CRISPR-Cas system has been applied to more than 40 species of ecologically diverse filamentous fungi and oomycetes, there has been little systematic analysis of the full spectrum of on-target DNA mutations (Liu et al. 2015, Nodvig et al. 2015, Schuster and Kahmann 2019). This is not surprising given that most of these studies used CRISPR-Cas to generate gene mutations for further functional characterization, and therefore did not catalog or explore specific DNA mutations or the involvement of different DNA repair pathways (Schuster and Kahmann 2019, Wang and Coleman 2019). Despite the lack of systematic analysis, unexpected on-target DNA mutations following CRISPR-Cas editing in filamentous fungi have been reported. In the human pathogen *A. fumigatus*, frequent insertions of linearized plasmid DNA (up to 6.5 kb) were found at the Cas9 targeting region (Fuller et al. 2015). In the plant pathogenic fungus, *Sclerotinia sclerotiorum*, amplification-based genotyping unexpectedly found that 100% of mutant phenotype transformants were PCR-negative using a primer pair amplifying across the Cas9 targeting site. Further TAIL-PCR and short-read sequencing revealed all of the transformants contained large plasmid DNA insertions, that importantly, lacked long stretches of homologous sequence to the genome (Li et al. 2018). Similar observations, reporting unexpected plasmid or foreigner DNA integration at Cas9 or Cas12a targeting sites, have been reported in other filamentous microbes, including *T. reesei, Phytophthora infestans, Nodulisporium* sp, and encapsulated yeast *C. neoformans* (Zheng et al. 2017, Hao and Su 2019, Yadav et al. 2020, Ah-Fong et al. 2021) (Fig. 3B). In fission yeast, insertion of carrier DNA from chum salmon (*Oncorhynchus keta*) has been found to integrate at the Cas9 cleavage site (Longmuir et al. 2019). There has been postulation that the observed large DNA integrations resulted from C-NHEJ, but experimental evidence was not provided (Fuller et al. 2015, Zheng et al. 2017, Li et al. 2018). In addition to foreign DNA integration, Cas9 gene editing in *T. reesei* resulted in tandem duplications at the targeted locus, which is a type of DNA repair profile that has been linked to C-NHEJ (Schimmel et al. 2017, Hao and Su 2019). Our lab recently reported detailed characterization of DNA repair events following Cas12a ribonucleoprotein (RNP) editing in the fungal plant pathogen, *M. oryzae*, that causes blast disease on rice and other monocots (Zhong et al. 2016, Peng et al. 2019, Valent 2021). This analysis revealed large donor DNA insertions as concatemer fragments of up to ∼20 kb at the Cas12a targeted site (Huang et al. 2021, Huang and Cook 2022). In addition to large insertions, we identified four other DNA repair outcomes in the presence of DNA donor- (i) simple insertion; (ii) large deletions; (iii) deletion plus insertions; and (iv) INDELs (Fig. 3B–D). A substantial number of independent transformants were analyzed using sanger sequencing and long-read sequencing and assembly, and the spectrum of observed DNA mutations suggested that multiple DNA repair pathways, including C-NHEJ, MMEJ, and SSA were involved (Huang et al. 2021). For example, multiple large deletion mutants at one edited locus were found to results from the combination of repetitive flanking DNA into a single repeat, which is thought to be a common outcome of SSA (Bhargava et al. 2016, Huang et al. 2021). For other transformants, the integration junctions of simple and large insertion mutants often contained microhomology between the insertion and genomic sequence, indicating the involvement of microhomology during DNA repair. The use of microhomology (∼50 bp) for precise integration of exogenous templates during Cas-induced DSB repair has been reported in other fungal species (Zhang et al. 2016, Al Abdallah et al. 2017, Foster et al. 2018, Leisen et al. 2020, Lax et al. 2021). The importance of homologous sequence for DSB repair also extends to a protozoan parasite that frequently underwent large deletions between two long-homology sequences during DSB repair (Zhang and Matlashewski 2019). As *Leishmania* lacks the known ligase involved in C-NHEJ (i.e. Lig4), the authors indicate that SSA is that most likely pathway responsible for generating these mutations (Zhang and Matlashewski 2019).

Prior to the CRISPR era, induced DNA repair was shown to be connected to specific repair pathways. A strategy termed Restriction Enzyme Mediated Integration (REMI) created DNA DSBs using restriction enzymes (Kahmann and Basse 1999). Using this approach, co-transferred DNA donor could be integrated at induced DSB sites, increasing transformation efficiency and the ratio of single-copy events (Kahmann and Basse 1999). Further genetic study revealed that Rad50, the core component of the MRX complex, was required for REMI in budding yeast, but Rad51 and Rad52 where dispensable (Schiestl et al. 1994). These results indicate that REMI might require the normal functioning MRX complex, which we can now infer is presumably dependent on MMEJ repair. It is also clear that the rate of HR mediated DNA DSB repair is not the same across species of filamentous pathogens. For instance, HR frequently results in highly efficient gene deletions in *Fusarium graminearum*, the causal agent of Fusarium head blight, while HR is infrequently observed in the maize pathogen *F. verticillioides*. (Choi and Shim 2008, Wang et al. 2011). To artificially increase the chances of HR as a means to improve functional genomics, mutant strains have been created that lack C-NHEJ (e.g. Ku70 or Ku80), with the rationale that if C-NHEJ is blocked then HR frequency will increase (Ninomiya et al. 2004). This method was first applied in *N. crassa*, and subsequently used in other filamentous pathogens, such as plant pathogens, *M. oryzae, Botrytis cinerea* and animal pathogens, *A. fumigatus*, and *Trichophyton mentagrophytes* (Ninomiya et al. 2004, da Silva Ferreira et al. 2006, Choquer et al. 2008, Villalba et al. 2008, Yamada et al. 2009). This approach has proven successful at increasing the rate of donor integrations, with one report showing inhibition of C-NHEJ repair led to 100% of fungal transformants containing the desired HR integrations (Ninomiya et al. 2004).

The described results establish that DNA editing in fungi and oomycetes, similar to animals, is tightly regulated by DSB repair pathways that interact, and the pathways have different propensities for on-site DNA mutations following induced DSBs. Future research is needed to determine the molecular details of the DNA DSB repair pathways in filamentous fungi, including their hierarchy and regulation, and ultimately the functional DNA mutation outcomes resulting from the different pathways. This can help resolve inconsistencies in the literature, such as Cas-based DNA integration using short homologous sequence being referred to as both MMEJ repair (Zhang et al. 2016, Al Abdallah et al. 2017, Foster et al. 2018) and HR (Lax et al. 2021). Further efforts are needed to test if knowledge from yeast are relevant in filamentous microbes, such as the involvement of two DNA polymerases, Pol δ and Pol λ, in MMEJ (Meyer et al. 2015). Additional areas of interest include understanding why there are two polymerases involved in MMEJ in yeast, versus one for TMEJ in animals and plants and the functional impact. Also, given the important role of Lig3 and PARP1 for TMEJ, do fungi contain analogous components during MMEJ given these two proteins are absent in fungi. Answering these questions will provide a deeper evolutionary and mechanistic understanding of DNA repair across eukaryotes.

## The contribution of DNA repair pathways to genome evolution in filamentous pathogens-the two-speed genome observation

Filamentous plant pathogens must overcome host immunity to cause disease and reproduce. We note that other plant-associated microbes, such as mutualists and endophytes, also counter the plant immune response in a similar manner to pathogens, but our review is pathogen-centric (Brader et al. 2017, Snelders et al. 2022). The plant innate immune system is largely characterized by receptor proteins that detect molecular signatures of the invading pathogen or the infection process through varied mechanisms (Cook et al. 2015). Plant immune receptor proteins either reside in the plasma membrane, typified by receptor-like kinases and receptor-like proteins (DeFalco and Zipfel 2021), or they are located in the cytoplasm, characterized as nucleotide-binding leucine-rich repeat/NOD-like receptor (NLR) proteins (Saur et al. 2021). While mechanistic details vary across pathosystems, a key paradigm is that successful pathogens employ a range of secreted proteins and small molecules, termed effectors, that suppress the plant immune system, modulate plant physiology, and protect the invading microbe (Oliveira-Garcia and Valent 2015, Snelders et al. 2022). Thus, the evolutionary stage is set for plant-microbe co-evolution, where selection for immune receptor recognition of pathogen effectors and signatures of infection imposes selection for receptor evasion or effector based immune suppression. Plant-microbe interactions can promote adaptive genome evolution, whereby the continued engagement of specific plant-microbe species produces refined mechanisms for both resistance and pathogenicity (Moller and Stukenbrock 2017, Sanchez-Vallet et al. 2018).

To better understand pathobiology and genome evolution, significant effort has been made to sequence and assemble filamentous pathogen genomes, including whole-genome sequencing of the rice blast causing fungus *M. oryzae* in 2005, and a number of other important model pathogens obtained through increasingly improved technology (Dean et al. 2005, Kamper et al. 2006, Tyler et al. 2006, Haas et al. 2009, Ma et al. 2010, de Jonge et al. 2013, Peng et al. 2019). Early analysis of the *P. infestans* genome led to a novel and insightful observation, where by the authors described the occurrence of gene-sparse regions that contain protein coding sequences with elevated levels of presence/absence variation, directional selection, and other features of adaptation to host infection (Raffaele et al. 2010). The authors named this phenomenon the ‘two-speed genome’ configuration, and numerous subsequently genomic studies of filamentous pathogens have described similar observations (Dong et al. 2015, Faino et al. 2016). The exact definition of the two-speed genome is vague, but is often characterized as the occurrence of (i) slow-evolving genomic regions coding for proteins with essential functions and sparse transposable element density, and (ii) fast-evolving regions coding for proteins associated with environmental or adaptive functions (e.g. host infection) and dense transposable element sequences. It is important to acknowledge that while the two-speed genome concept is an attractive generalization, it appears that some fungal phytopathogens do not display strong signals of genome compartmentalization (Frantzeskakis et al. 2018, Stam et al. 2018). As with many black and white dichotomies of biological systems, fungal pathogen genomes likely display a continuum of organizations with respect to gene and TE densities and the presence of variation (Frantzeskakis et al. 2019, Torres et al. 2020).

### Refining the description of the two-speed genome and associated characteristics

One aspect that needs further consideration is what exactly constitutes the two-speed genome. The two-speed genome classification has involved different metrics across filamentous pathogens. One common description has been the equation of two-speeds with the occurrence of lineage-specific (LS) and core genomic regions. Here, LS regions are defined by the presence/absence of DNA sequences between different strains of often the same species. LS regions identified from the blast fungus *M. oryzae*, and vascular wilt pathogen *Verticillium dahliae* are enriched in repetitive elements and *in planta* expressed genes (de Jonge et al. 2013, Bao et al. 2017). Subsequent study in *V. dahliae* refined the description of LS regions to include features of chromatin and transcription, and found these updated genomic regions better correlated with characteristics of host infection (Cook et al. 2020). The genomic regions appear enriched for coding sequences that are not essential but involved in specific life-history events, and therefore were renamed as Adaptive Genomic Regions (Cook et al. 2020). Another description of the two-speed genome is based on the occurrence of AT-rich blocks, called AT-rich isochores, defined by base pair composition divergence at distinct regions compared to genome or species wide averages. In the phytopathogenic fungus, *Leptosphaeria maculans*, AT-rich isochores contain ∼33% GC content and are over-represented for sequences coding effectors (Rouxel et al. 2011). Another important feature of filamentous pathogen genomes that can contribute to a two-speed genome is the occurrence of dispensable chromosomes, also called mini-chromosomes, supernumerary chromosomes, accessory chromosomes or A/B chromosomes (Coleman et al. 2009, Galazka and Freitag 2014, Peng et al. 2019). The occurrence of dispensable chromosomes has been documented across diverse plant, animal, and microbial systems, sometimes without a clear phenotypic or evolutionary impact (D'Ambrosio et al. 2017, Soyer et al. 2018, Peng et al. 2019). In fungal pathogens, seminal work and subsequent experimentation has clearly demonstrated the importance of dispensable chromosomes in the evolution of virulence of *Fusarium* species (Ma et al. 2010, Li et al. 2020). There is also strong evidence that dispensable chromosomes contribute to virulence and evolution in the blast fungus *M. oryzae* (referred to at present as mini-chromosomes in *M. oryzae*) (Chuma et al. 2011, Peng et al. 2019, Langner et al. 2021, Liu et al. 2022). Indeed, previous consideration of dispensable chromosomes detailed how these regions offer fungal pathogens a genomic ‘cradle’ possibly accelerating pathogen evolution (Croll and McDonald 2012). Quantifying rates of adaptive evolution is challenging, but it is clear that dispensable chromosomes can be quite dynamic, even under axenic lab growth (Moller et al. 2018).

These various descriptions of the two-speed genome have not used a common vernacular or criteria, instead the characterization has been based on sequence conservation, content, or physical arrangement, while the original categorization in *P. infestans* was based on gene density (Raffaele et al. 2010). What appears common between the descriptions is that many filamentous pathogen genomes have a non-uniform distribution of coding sequences related to host infection, and these coding sequences show elevated rates of sequence variation (e.g. Single-Nucleotide Polymorphisms (SNPs), copy number variation (CNV), presence/absence variation (PAV)) (Raffaele et al. 2010, Rouxel et al. 2011, de Jonge et al. 2013). More simply, many fungal pathogen genomes have segments enriched for non-essential coding sequences that are highly variable. We suggest that while individual experimental details or analysis may have emphasized the occurrence of gene-sparse, AT-rich, LS regions, or dispensable-chromosomes, the unifying theme is segments of heightened variation. This may lead to accessory chromosomes or accessory regions of pathogen genomes in a population (Moller and Stukenbrock 2017). There are likely biological differences between filamentous fungal pathogens, such as reproductive mode, ploidy, genome size, and repeat content that influence the exact manifestation of a compartmentalized genome. Further research and theory are needed to understand if differences in genome compartmentalization, such as gene-sparse, isochores, LS regions or dispensable-chromosomes, differentially impact fungal fitness and evolutionary potential. Alternatively, the different genome configurations may reflect that there are multiple routes to answering evolutions challenge of maintaining fitness in a changing environment. Under the idea of the latter, dispensable chromosomes are a more extreme example of compartmentalized (i.e. non-uniform) genome organization compared to LS regions for example, but they both ultimately represent regions of the genome with a different evolutionary trajectory compared to regions outside the dispensable or LS regions. Interestingly, these observations are not restricted to plant filamentous pathogens, as there are recent reports of the similar bipartite genome composition in the animal pathogens *Batrachochytrium dendrobatidis* (*Bd*) and *B. salamandrivorans* (Wacker et al. 2021) and the model arbuscular mycorrhizal fungus *Rhizophagus irregularis* (Yildirir et al. 2022).

### A unifying cause for the two-speed genome

This leads to the bigger question of what accounts for the formation and maintenance of gene-sparse, LS or dispensable chromosomes? Are these configurations driven only by selection, or are there additional intrinsic genomic mechanisms that contribute to this configuration? This is an important question for the two-speed genome observation, which currently lacks systematic understanding and clear testable hypotheses ultimately needed to advance our understanding of pathogen genome evolution (Torres et al. 2020). We propose two competing hypotheses for the creation and maintenance of the two-speed genome, a passive selection alone model, and an active mechanistic model termed Biased Variation.

Under the selection alone model, DNA variation (e.g. SNPs, INDELs, duplications, inversions) arises at a nearly uniform rate across the genome. Most sequence variation would be evolutionarily neutral, but variation in essential coding sequences with lethal or fitness reducing effects would be selected against and removed from the population (i.e. purifying selection). Variation that arises in regions with less selective constraints, such as regions contributing to specific environmental conditions or non-essential functions, would remain in the population for a longer period of time, and could eventually increase fitness under specific environmental conditions. This description of conventional evolutionary theory can explain present-day genome configuration whereby essential coding sequences have relatively less sequence variation in a population than regions contributing to non-essential or adaptive functions. This model does not evoke any special status or rules for the two-speed genome, and any apparent correlations seen today are non-causative associations.

**Figure 4.**
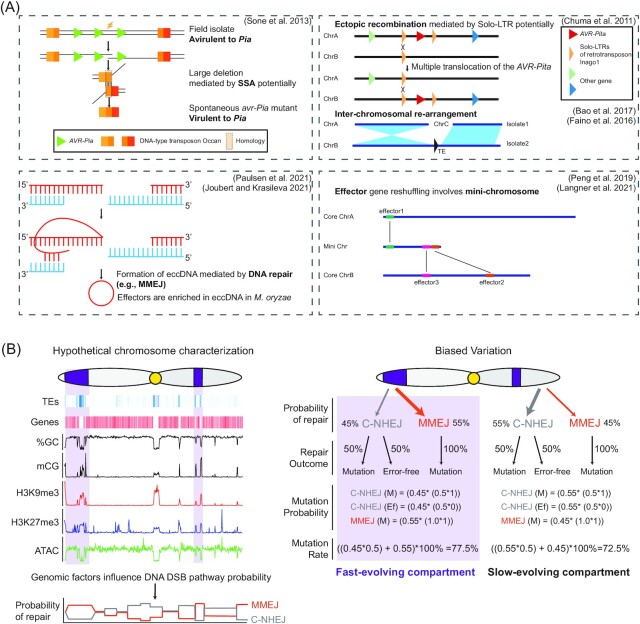
The association among DSB repair, genome compartments and biased genome evolution in filamentous pathogens. **(A)** Examples of observed genome variation influenced by DNA repair. Transposable element (TE) or other repeated DNA can mediate ectopic recombination or re-arrangements (top). DNA contained outside of the core genome, such as eccDNA and mini- or dispensable-chromosomes, may arise due to DNA repair mechanisms, or shuffle DNA with core chromosomes, contributing to biased genome variation in filamentous pathogens (bottom). **(B)** Illustration of a hypothetical chromosome characterized for multiple genomic, epigenomic and chromatin variables. These factors may collectively influence the DNA repair hierarchy at a given genomic locus. We provide a specific example of how DNA repair choice between C-NHEJ and MMEJ could lead to different rates of DNA mutation in the genome, a model we refer to as Biased Variation. Note, half of the DNA DSBs repaired by C-NHEJ may not cause any DNA mutation, which we represent as 0 in the mutation probability calculation, while half and all DNA DSBs repaired by C-NHEJ and MMEJ will results in a DNA mutation, represented by a 1 in the mutation probability calculation. The rates and frequencies used here are approximations and remain to be experimentally determined. (M), mutation; (Ef), error-free.

A competing hypothesis to the selection alone model attempts to incorporate numerous described associations between genomic features and the two-speed genome. There have been proposed links between the two-speed genome and the epigenome, owing to the unique chromatin features and composition of repetitive elements in dynamics compartments of the genome (Faino et al. 2016, Seidl et al. 2016, Cook et al. 2020, Torres et al. 2020, Chen et al. 2022), but a single, clear mechanism has remained elusive. We summarize a few associations here and propose the ‘Biased Variation’ model as a means that could create a two-speed genome. An important association is the co-occurrence of TEs and structural variation at distinct regions of the two-speed genome (de Jonge et al. 2013, Faino et al. 2016, Moller and Stukenbrock 2017). The first sequence and assembly of a *M. oryzae* mini-chromosome identified sequence similarity between the ends of some core-chromosomes and the mini-chromosome (Peng et al. 2019). This sequence similarity and enrichment for TEs and copy-number variation may mediate effector re-shuffling, providing an adaptive benefit (Fig. 4A). This was speculated to occur in *M. oryzae* based on observations of the first cloned avirulence effector from *M. oryzae, AVR-Pita* (Orbach et al. 2000). Two functional members in *AVR-Pita, AVR-Pita1* and *AVR-Pita2*, were found located on different chromosomes across multiple isolates, while the non-functional member *AVR-Pita3* was fixed at one location. Sequence of a retrotransposon element, termed Inago1, was found flanking all tested *AVR-Pita1/2* loci, and thus ectopic recombination mediated by Inago1 was considered a potential mechanism for the frequent translocation of *AVR-Pita1/2* (Chuma et al. 2011) (Fig. 4A). Error-prone DNA DSB repair is also proposed to be involved in the formation of LS regions through transposon mediated genome recombination in *V. dahliae* (Faino et al. 2016) (Fig. 4A). In addition to translocations, transposable elements can influence genome variation through SSA mediated deletion, such as the spontaneous deletion of 44 kb between flanking copies of the transposon *Occan*, which resulted in deletion of three copies of the avirulence effector *AVR-Pia* (Sone et al. 2013) (Fig. 4A). In addition to plant pathogens, copy number variation and loss of heterozygosity were proposed to result from SSA repair using long repeat sequences in the human pathogen *Candida albicans* (Todd et al. 2019). As such, there are clear occurrences of DNA repair mediated mechanisms at repetitive sequences resulting in significant genome variation for important loci in filamentous pathogens (Seidl and Thomma 2017). In addition to these observations, a clear association between the epigenome and dispensable chromosome has been found in the wheat pathogen *Z. tritici*. Enriched histone modification of H3K27me3 on dispensable chromosomes is a distinguishing feature when compared to core chromosomes (Schotanus et al. 2015). Connecting to the previous sections of this review, the MMEJ repair pathway, which creates more mutagenic outcomes than C-NHEJ and HR, has been reported to be more activated at H3K27me3 marked heterochromatin (Her and Bunting 2018, Schep et al. 2021). This would be consistent with the observed increase in genomic instability and increased mutation rate at H3K27me3 regions in *Z. tritici* (Moller et al. 2019, Habig et al. 2021). Histone methylation H3K36me3 by Ash1 has also been proposed to effect DNA repair and genome stability in the rice pathogen *F. fujikuroi* (Janevska et al. 2018). Therefore, it is reasonable to speculate that DNA repair, the epigenome, and chromatin status are actively involved in the formation and maintenance of the two-speed genome. We propose the ‘Biased Variation’ model, whereby the creation of DNA variation is suppressed or enhanced at specific genomic regions, which is subsequently subject to natural selection.

### Biased Variation model: synthesis of the epigenome, chromatin, and DNA repair as a mechanism for biased genome evolution

The ‘Biased Variation’ model proposes that variation for genomic factors influence DNA repair preferences and leads to mutational bias in the genome. That is, genome variation arises at unequal rates prior to selection. DNA mutation does not arise uniformly in the genome, but rather follows local probability functions for the creation of DNA mutation. It is important to note, this model does not suggest that the rise of DNA mutation is influenced by the environment or that it co-occurs with selection. The famous Luria-Delbruck fluctuation test demonstrated that DNA mutation arises pre-selection (Luria and Delbruck 1943). As discussed in this review, features such as base pair sequence content and repetitive DNA density, chemical modifications to DNA and histones, and nuclear DNA arrangement have all been implicated to influence DNA repair. As there are multiple DNA repair pathways that show variation in their mutation profiles, and specific genomic features influence the preference for specific DNA repair pathways, the interplay between DNA repair, the epigenome and chromatin could explain the occurrence of the two-speed genome (Fig. 4B). As a simple illustration of this model, we focus on only two DNA DSB pathways, C-NHEJ and MMEJ. The C-NHEJ DSB pathway can result in either perfect repair, resulting in no detectable DNA mutation (i.e. error-free), or imperfect repair (i.e. mutation), resulting in altered sequence (i.e. INDEL) (Fig. 4B). Experimental evidence supports that high-fidelity C-NHEJ is possible, but to our knowledge there is not a clear estimate of this rate, certainly not in filamentous fungi (Betermier et al. 2014). We therefore use 50% chance for either perfect or imperfect C-NHEJ repair as an approximation. For DNA DSBs repaired by MMEJ, all resulting loci will have a DNA mutation due to end resection and flap trimming. There is clear evidence for DNA DSB pathway preference, but the exact quantification is currently not clear. We therefore assume a 5% deviation from equal repair pathway preference at certain regions of the genome for MMEJ over C-NHEJ, 55% versus 45% respectively (Fig. 4B). This simple probability difference in DNA repair pathway preference, coupled with the probability to create a DNA mutation, results in an estimate that genome regions preferentially repaired by MMEJ will experience 5% more DNA mutations (Fig. 4B). While a 5% increase in DNA mutation may seem modest, at least two factors in filamentous pathogen biology make this increase substantial. One fact is that fungi do not have a described separation between germline and somatic tissue. Theoretically, any somatic tissue can give rise to asexually produced spores, and therefore any somatic mutations could be heritable. This significantly increases the number of cells that could undergo a heritable mutation and therefore increases their frequency. Additionally, experimental evidence in animal models show that germline cells have lower rates of mutation compared to somatic tissue (Milholland et al. 2017). It is interesting to speculate that fungi may have comparatively elevated genomic instability compared to metazoans for instance because they lack germline separation. The second related piece is the incredible volume of progeny that microbes, including filamentous pathogens, release with respect to both absolute time and growth normalized time. The number of progeny, and therefore the number of individuals effected by Biased Variation, are orders of magnitude greater in microbes than animals or plants. As such, the biology of filamentous pathogens could result in Biased Variation having an increased impact on their genome evolution compared to organisms in other domains of life.

Our simple example of Biased Variation using differences for C-NHEJ and MMEJ repair preference and mutation rate highlight the potential impact on genome evolution. This example ignores many other variables, such as the impact of other DNA repair pathways and genomic variables that influence their activity and mutational profiles. Future research is needed to unpack these associations and generate estimates for their interactions. Mutation accumulation experiments in both a fungal plant pathogen and the model dicot *Arabidopsis*, showed a clear association between the observation of accumulated DNA variation and components of the epigenome (Habig et al. 2021, Monroe et al. 2022). Re-analysis of fast neutron induced mutations in a population of rice (Li et al. 2017) identified a significant reduction in mutation probability for genic and non-genic regions associated in or around H3K4me1 domains (Quiroz et al. 2022). Such findings are consistent with the Biased Variation model, whereby the histone modification H3K4me1 could influence the propensity of HR DSB repair in marked regions, providing higher fidelity repair. It is interesting to note that research on the topic of biased genome variation (i.e. intragenomic mutation variability) in filamentous pathogens has tended to focus on regions and causes of hypervariable genomic regions (Coleman et al. 2009, Raffaele et al. 2010, de Jonge et al. 2013, Dutheil et al. 2016, Cook et al. 2020), while recent research in plants has described the occurrence of hypovariable genomic regions (Monroe et al. 2022, Quiroz et al. 2022). It will be interesting to bridge these results, and investigate the occurrence of such mechanisms across domains of life, to understand how DNA repair hierarchy and regulation contribute to hyper- and hypovariable genomic compartments, and ultimately how such processes impact genome evolution.

In addition to genomic factors that influence DNA DSB repair preference, the occurrence of DNA DSBs themselves are likely not uniform across the genome. Genomic factors such as repetitive sequence density, transcriptional activity, and DNA replication timing interact and influence the creation of DNA DSBs (Hamperl and Cimprich 2016, Gadgil et al. 2017, Sebastian and Oberdoerffer 2017). This would lead to regions of the genome being more prone to DNA DSBs, and therefore more impacted by Biased Variation for DNA repair. This would be consistent with the fact that multiple characterizations of faster evolving sections of filamentous pathogen genomes involve regions with higher TE densities (Faino et al. 2016, Moller and Stukenbrock 2017). In addition to TEs serving as homologous regions to anchor DSB repair, TEs can also impacting the creation of DNA variation through active transposition that may further contribute to Biased Variation (Fouche et al. 2022).

Another factor we speculate will have importance in Biased Variation is the creation and function of extrachromosomal circular DNAs (eccDNAs). Previous studies in eukaryotes have detailed adaptation to stress through amplifying gene content on eccDNAs. For example, the herbicide target gene coding 5-enolpyruvlyshikimate-3-phosphate synthase can be amplified in the form of eccDNAs and heritably transmitted, thus causing herbicide resistant in the agronomically important weed *Amaranthus palmeri* (Koo et al. 2018). Interestingly, a recent pre-print using whole-circularome sequencing in *M. oryzae* revealed that eccDNA-associated genes have significant longer distance to the nearest gene but shorter distance to the nearest repeats than other genes, which suggests these eccDNA-producing regions are located in fast-evolving genomic compartments (Joubert and Krasileva 2021). Indeed, effector coding sequences are enriched in the production of eccDNAs (Joubert and Krasileva 2021) (Fig. 4A). Interestingly, a recent study in human cell lines found that the formation of eccDNAs is facilitated by DNA DSB and dependent on the balance between C-NHEJ and the TMEJ pathway (Paulsen et al. 2021). As an important downstream component of TMEJ pathway, Lig3 is also found to be required for the generation of eccDNAs (Wang et al. 2021). Future research determining how mechanisms of DNA repair pathway choice and their resulting mutational profiles are influenced by the epigenome and chromatin will provide a comprehensive understanding of first principles affecting genome function and genome evolution.

## Concluding remarks and future perspectives

The repair of DNA DSBs is a well-studied and critical function of eukaryotic genomes. Multiple repair pathways have evolved to fix DNA DSBs, likely highlighting their importance and the need for redundant mechanisms, but the use of multiple systems may also highlight levels of specialization and unique outcomes of the different pathways. Here we have summarized mechanistic details of four DNA DSB repair pathways with an emphasis of what is known or remains unclear in fungal and oomycete pathogens. It is clear that a number of physiological, molecular, and biochemical factors influence DNA DSB repair choice for a given break. The use of CRISPR-Cas, or other genome editing strategies that rely on DNA DSB creation and repair are subject to these rules, and influence resulting outcomes. Naturally occurring DNA DSBs are equally influenced by the interconnection and hierarchy of DNA DSB repair pathways and epigenetics and chromatin states. We describe here a model, termed Biased Variation, and provide a simple probabilistic example demonstrating how genomic factors could influence DNA repair choice and impact the creation of DNA variation. The model is an attempt to understand mechanisms that create unequal DNA variation in the genome. Future research is needed to expand and test the Biased Variation model, or generate other mechanistic models of observed compartmentalized genome evolution. Further developing the understanding of how DNA repair influences genome stability and evolution will impact our ability to engineer genomes and predict evolution related to industrial, clinical, and agricultural aspects of oomycetes and fungi.
